# Infection of non-cancer cells: A barrier or support for oncolytic virotherapy?

**DOI:** 10.1016/j.omto.2022.02.004

**Published:** 2022-02-12

**Authors:** Victor A. Naumenko, Aleksei A. Stepanenko, Anastasiia V. Lipatova, Daniil A. Vishnevskiy, Vladimir P. Chekhonin

**Affiliations:** 1 V. Serbsky National Medical Research Center for Psychiatry and Narcology, Moscow 119034, Russia; 2 Department of Medical Nanobiotechnology, N.I Pirogov Russian National Research Medical University, Moscow 117997, Russia; 3 Center for Precision Genome Editing and Genetic Technologies for Biomedicine, Engelhardt Institute of Molecular Biology, Russian Academy of Sciences, Moscow 119991, Russia

**Keywords:** oncolytic virus, infection of non-cancer cells, cancer therapy, tumor microenvironment, secondary lymphoid tissues

## Abstract

Oncolytic viruses are designed to specifically target cancer cells, sparing normal cells. Although numerous studies demonstrate the ability of oncolytic viruses to infect a wide range of non-tumor cells, the significance of this phenomenon for cancer virotherapy is poorly understood. To fill the gap, we summarize the data on infection of non-cancer targets by oncolytic viruses with a special focus on tumor microenvironment and secondary lymphoid tissues. The review aims to address two major questions: how do attenuated viruses manage to infect normal cells, and whether it is of importance for oncolytic virotherapy.

## Graphical abstract

**Figure:**
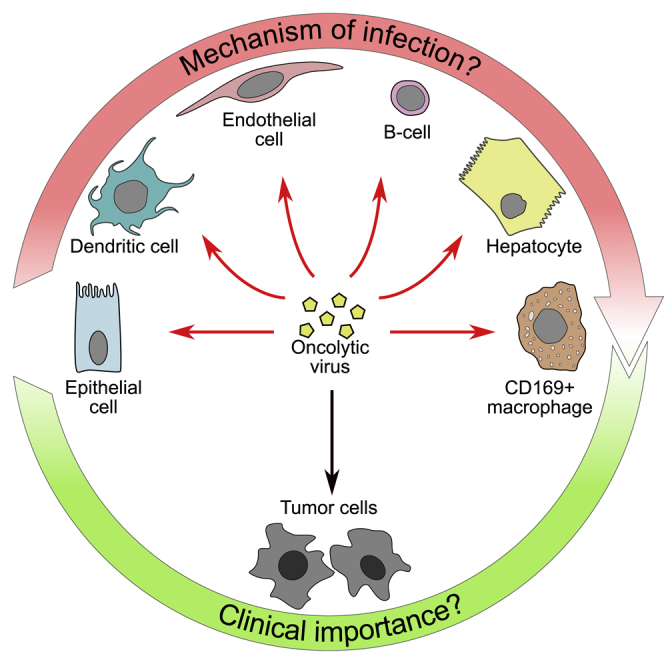

## Teaser

Multiple normal cells are sensitive to oncolytic viruses, although at a greatly reduced rate compared with cancer cells. The off-target infection may play dual roles by stimulating antitumor responses or causing toxicity. Deciphering the mechanisms responsible for normal cell infection will help improve the efficacy and safety of the therapy.

## Introduction

Oncolytic virotherapy (OVT) is at the cutting edge of the current cancer research efforts. Using a virus as a specific tool to find and destroy cancer cells while sparing normal tissues is at the heart of the OVT paradigm. While some viruses are oncotropic by nature, the majority of oncolytic viruses (OVs) are designed to enhance tumor targeting and ensure their safety for the host. To selectively restrict OV activity to the tumor cells, multiple strategies have been applied including (1) transcriptional targeting by using tumor- or tissue-specific promoters; (2) genetic or chemical modification of viral capsid for selective binding to the receptors upregulated in cancer cells; (3) alterations of viral genes incompatible with OV replication in normal cells, but compensated in malignant cells due to impaired biochemical pathways; and (4) microRNA-based regulation of viral gene expression.^1^^,^^2^

To date, the most encouraging results of OVT are associated with intratumoral (i.t.) administration that provides high and local OV concentration in the tumor and limits off-target effects. However, this approach is restricted to the tumors that are directly accessible for injection. Systemic administration of OVs has the potential to expand the applicability of OVT to cancers with various locations and histologies, including metastases. At the same time, intravascular delivery of OVs inevitably broadens the range of host cells that interact with a virus, leading to suboptimal delivery to the tumor sites and increasing the risk of side effects. It should be noted that billions of viral particles injected into the blood flow is far above the viral loads observed during natural infections. Although the majority of the viruses used as a platform for designing OVs are not blood-borne pathogens and/or do not normally infect humans, a high bolus dose could potentially overcompete with antiviral host defense programs.

Despite the generally good tolerability of OVs in clinical trials, multiple biodistribution studies demonstrate that OVs can replicate to some extent in non-cancer cells. We believe there are at least two reasons to discuss this topic in detail. First, under certain conditions, infection of normal cells may compromise therapeutic efficacy and cause serious side effects. On the other hand, transient amplification of viral antigens in the host cells may alert the immune system to the previously tolerated tumor. In this regard, OV infection of the tumor microenvironment (TME) and secondary lymphoid tissues is of special interest.

Here we review the reports on OV infection of non-cancer cells, mainly focusing on the viruses tested in clinical trials for systemic treatment. For each virus, the attenuation strategies are briefly described, followed by the discussion of the mechanisms enabling OV replication in normal cells and potential implications of this infection for OVT.

## How do OVs manage to infect non-cancer cells?

### Poxviruses

Currently, vaccinia virus (VV), a member of *Poxviridae* family, is among the most prominent OV platforms showing encouraging results in clinical trials.^1^ VV strains are known to inherently target tumors. This natural selectivity is mostly based on the activation of the epidermal growth factor receptor (EGFR) pathway in malignant cells driving VV replication.^3^ Moreover, low tumor cell responsiveness to interferon (IFN) makes them susceptible to infection, in contrast with normal cells that are protected by antiviral cytokines.^4^ To further increase tumor-specific targeting and safety for normal tissues, several attenuated VV mutants have been constructed. For instance, Pexa-Vec (JX-594), a Wyeth strain of VV, lacks the *J2R* gene, encoding viral thymidine kinase (TK), and its replication is restricted to cancer cells with enhanced TK activity.^5^^,^^6^ Similarly, vvDD (JX-929) was developed by deleting *J2R* and *C11R* (vaccinia growth factor) genes in a Western Reserve strain of VV.^7^ Attenuation of GL-ONC1 (GLV-1h68), an OV based on Lister VV strain, is achieved by disruption of TK, hemagglutinin, and *F14.5L* genes.^8^ Although these vectors demonstrate good safety and tolerability in clinical trials, there is a growing body of literature showing infection of non-cancer cells by oncolytic VV strains (Table 1).

**Table 1:** *In vivo* infection of non-cancer targets by OVs

Reference	Virus	Treatment	Tumor	Time point	Target cell/organ	Mechanism of infection	Role of infection
**Poxviruses**							

Kirn et al.^9^	WR-delB18R	1E8 PFU i.v.	JC, CMT-93	24 h	tumor EC	NS	antiangiogenic effect
Ottolino-Perry et al.^10^	vvDD-SR	1E9 PFU i.p.	MC38	3 d, 6 d	tumor EC	NS	antiangiogenic effect
Arulanandam et al.^11^	vvDD	1E9 PFU i.v.	MC38	72 h	tumor EC	VEGF suppresses IFN response	NS
			skin wound	72 h	EC		
Breitbach et al.^12^	WR-TK(-)	1E8 PFU i.v.	4T1, MC38	48 h	tumor EC	VEGF and FGF-2 sensitize EC to OV infection	antiangiogenic effect
	JX-594	≥1E9 PFU i.v.	Patients’ tumors	7 d	tumor EC		
Kim et al.^13^	mpJX-594	1E7 – 5E7 PFU i.v.	RIP-Tag2, U87	6 h, 1 d, 5 d	tumor EC	NS	loss of EC barrier function
				6 h	spleen	NS	NS
Autio et al.^14^	vvDD-hCD40L	1.2E8 TCID_50_/kg	no tumor (dogs)	24 h	spleen (macrophages)	NS	NS
Béguin et al.^15^	TG6002	1E7 PFU/kg	no tumor (dogs)	35 d	spleen	NS	NS
Tysome et al.^16^	LIVP	1E7 PFU i.v., 1E7PFU i.t.	Suit-2	24 h, 72 h, 5 d, 10 d, 20 d	spleen (monocytes)	NS	NS
Huang et al.^17^	GL-ONC1	2E6 PFU i.v.	A549	14 d	spleen, lungs	NS	NS
Yu et al.^18^	GL-ONC1	1E6 PFU i.v.	PANC-1	14 d, 28 d	ovaries, spleen, lungs	NS	NS
Hofmann et al.^19^	GL-ONC1	1E7 PFU i.v.	PC14PE6	7 d, 14 d	ovaries	NS	NS
Kim et al.^20^	JX-594	1E9 PFU i.v.	VX2 (rabbits)	24 h, 48 h	ovaries	NS	NS
McCart et al.^21^	vvDD	1E7 PFU i.p.	MC38	8 d	ovaries	NS	NS
Jenne et al.^22^	MYXV	1E7 PFU i.v.	no tumor	8 h	liver (macrophages)	NS	NS

**HSV**							

Benencia et al.^23^	HSV-1716	5E6 PFU i.v., 5E6 PFU i.t.	ID8-VEGF	10 d	tumor EC	VEGF upregulates HVEM	antiangiogenic effect
Cinatl et al.^24^	G207	1E7 PFU i.t.	ARMS	24 h	tumor EC	RR overproduction in proliferative ECs allows for OV replication	antiangiogenic effect
Mahller et al.^25^	G207, hrR3	5E6 PFU i.t.	MPNSTs	7 d	tumor EC	NS	antiangiogenic effect

**Reovirus**							

Samson et al.^26^	Reolysin	1e10 TCID_50_ i.v.	patients’ brain tumors	3–17 d	tumor EC	NS	NS
Loken et al.^27^	Reolysin	1e7 PFU i.t.	MDA-MB-468	2–16 d	normal EC heart skeletal muscle	NS	myocarditis and vasculitis in immune-compromised hosts
Chakrabarty et al.^28^	Reolysin	6.5e9 TCID_50_ i.v.	no tumor	24 h, 72 h	spleen heart	NS	NS

**Rhabdoviruses**							

Breitbach et al.^29^	VSVΔ51	5E8 PFU i.v.	CT26	24 h	tumor EC	NS	antiangiogenic effect
Naumenko et al.^30^	VSVΔ51	5E8 PFU i.v.	CT26	8 h	SSM MMM splenic DC splenic B cells	NS	NS
Kim et al.^31^	VSVΔ51	1E8 PFU i.v.	EMT6	8 h	SSM MMMs	NS	NS
Bridle et al.^32^	VSVΔ51	1E9 PFU i.v.	B16	6 h	splenic follicular B cells	NS	boosting antitumor immune response
Breitbach et al.^33^	VSVΔ51	1E9 PFU i.v.	CT26	8 h, 24 h	spleen lung	NS	NS
Zhang et al.^34^	VSV-IFNβ	1E9 TCID_50_ i.v.	no tumor	3 h, 24 h	spleen	NS NS	NS NS
		1E6, 1E7, 1E8 TCID_50_ i.v.	5TGM1	48 h			
Leblanc et al.^35^	VSV-IFNβ	1E11 TCID_50_ i.v.	no tumor (dogs)	30 d	spleen	NS	NS
Pol et al.^36^	MG1	1E9 PFU i.v.	B16	24 h, 48 h	spleen	NS	NS
Pol et al.^37^	MG1	1E10, 1E11 PFU i.v.	no tumor (macaques)	11 d	spleen lymph nodes	NS	NS

**Measles virus**							

Jing et al.^38^	MV-Edm-uPAR	1.5E6 TCID_50_ i.v.	MD-MBA-231	72 h	tumor EC	VEGF upregulates uPAR expression in ECs	NS
		1E6 TCID_50_ i.t.	4T1	48 h			
Myers et al.^39^	MV-Edm-NIS	1E7 TCID_50_ i.v.	no tumor	2 d, 5 d, 22 d	spleen	NS	NS
Peng et al.^40^	MV-Edm	1E7 PFU i.v.	no tumor	48 h	MMMs	NS	NS

**Adenoviruses**							

Majem et al.^41^	AdΔ24RGD, ICOVIR-1, ICOVIR-2	1E12 VP/kg	no tumor	72 h	liver	NS	hepatotoxicity
Leja et al.^42^	Ad-CgA	5E10 VP i.v.	no tumor	6 h, 24 h, 48 h	liver	NS	hepatotoxicity
Machitani et al.^43^	TRAD	1E9 IFU i.v.	no tumor	48 h	liver	NS	hepatotoxicity
Marino et al.^44^	Enadenotucirev	5E9 VP i.v.	A549	1 d, 2 d, 4 d, 8 d	liver	NS	NS
Li et al.^45^	ICOVIR15	1.2E7 PFU i.t.	005 GBM	96 h	tumor pericytes	NS	NS
Franco-Luzón et al.^46^	MAV-1 dlE102	i.v.	TH-MYCN	7 d	spleen	NS	NS
Garcia-Carbonero et al.^47^	Enadenotucirev	0.6–3.0E11 VP i.t.	patients’ colorectal tumors	8–15 d	lymph node	NS	NS

SR, somatostatin receptor; TK, thymidine kinase; CgA, chromogranin-A; TRAD, Telomerase-specific replication-competent adenoviruses; i.v., intravenous; i.t., intratumoral; i.p., intraperitoneal; PFU, plaque-forming units; TCID_50_, 50% tissue culture infectious dose; VP, virus particles; ARMS, human alveolar rhabdomyosarcoma; MPNSTs, malignant peripheral nerve sheath tumors; EC, endothelial cells; SSM, subcapsular sinus macrophages; MMM, marginal metallophilic macrophages; DC, dendritic cells; VEGF, vascular endothelial growth factor; FGF2, fibroblast growth factor ; IFN, interferon; RR, ribonucleotide reductase; HVEM, herpesvirus entry mediator; OV, oncolytic virus; NS, not studied.

Infection of tumor endothelial cells (ECs) by VV was first shown by Bell's group in subcutaneous syngeneic mouse tumors.^9^ Later, the same authors broadened this observation to multiple cancers and oncolytic VV strains.^10^, ^11^, ^12^, ^13^ Importantly, EC infection with viral transgene expression was demonstrated in human tumor biopsies of diverse histologies.^12^ Enhanced susceptibility of human umbilical vein endothelial cells (HUVECs) to VV was also observed *in vitro* after cells' exposure to tumor-conditioned media or vascular endothelial growth factor (VEGF).^10^, ^11^, ^12^ The sensitivity of tumor vasculature to VV was initially linked with the enhanced proliferative and metabolic activity of ECs in newly formed vessels and/or overexpression of EGFR on these cells. More recently, it has been shown that the phenomenon is not VV specific and is mediated by increased levels of VEGF that suppress antiviral IFN response through the VEGF-A/VEGFR2/Erk/Stat3 signaling axis in tumor endothelium, rendering the cells susceptible to a range of OVs.^11^ VEGF-A is also known to facilitate VV entry and replication *in vitro* by activation of Akt pathway.^3^

It is not clear whether ECs may permit productive VV infection and contribute to viral spread in the tumor. While some studies report that endothelial infection starts as early as 6 h post injection (hpi)^13^ and precedes viral spread in the tumor core,^12^ others reveal vascular infection only at late time points when a significant part of the tumor is infected.^10^ Regardless of the initial source of viral infection in the tumor, the ability of oncolytic VV strains to target tumor endothelium and disrupt vasculature has been shown in multiple tumor models. However, the characterization of VV effect on the vasculature is not complete yet. The earlier studies have described a dramatic decrease in the vessel density within the tumors upon VV treatment both in preclinical models and in humans,^9^^,^^10^^,^^12^^,^^33^^,^^48^, ^49^, ^50^ but a more recent report argues against vascular collapse and identifies the loss of barrier function in infected ECs as the main vascular effect of VV.^13^

It should be noted, though, that the ability of VV to infect tumor ECs is not supported by Szalay's group.^17^^,^^19^^,^^51^^,^^52^ Studying virus distribution in human xenografts during a late phase of treatment (36–42 days), the authors demonstrated infection of the tumor cells, but not endothelium. These conflicting results may reflect the dynamics of EC infection that could be eliminated at later time points. It is also possible that, in a xenograft model, murine ECs are less responsive to human tumor signaling and therefore more resistant to VV infection. Importantly, even in the absence of EC infection, systemic VV treatment leads to a decrease in VEGF levels and densities of tumor blood vessels^17^^,^^19^ and lymphatic vessels.^52^ These results suggest that VV antiangiogenic effect is more likely mediated by cancer cell infection and subsequent downregulation of vessel supporting factors, rather than direct targeting of ECs. Consistent with this hypothesis, the loss of vascular perfusion and suppressed VEGF levels are observed throughout the period of active viral replication in the tumor.^53^ More precise investigation of VV spread in the tumors is required to uncover the mechanisms of the antiangiogenic effect and to assess the role of EC infection in vascular disruption.

The infection of ovaries by parental^54^, ^55^, ^56^ and attenuated VV strains^18^, ^19^, ^20^, ^21^ has been reported in rodents, rabbits, and non-human primates. Following intravenous (i.v.) injection, VV antigens are detected in the ovaries^54^ and viral titers persist in the organ for several weeks.^18^^,^^19^ Similar to tumors, the ovarian follicle is the site of actively replicating cells that are potential targets for VV. Moreover, upregulation of VEGF in perifollicular areas could sensitize ECs to infection and/or lead to increased vessel permeability and virus accumulation in the organ.^21^ These hypotheses need to be investigated as well as the potential ovarian toxicity of oncolytic VV strains.

Biodistribution studies suggest that limited VV replication is supported by splenocytes. Spleen and liver are the major filters for circulating pathogens and therefore the detection of VV in these organs may simply reflect the sequestration of the injected virus. Nevertheless, several independent studies demonstrate that VV is found more frequently and with higher titers in the spleen than in the liver,^15^, ^16^, ^17^, ^18^ indicating that splenocytes may permit viral replication. Although the infected cells have not been identified yet, it is likely that VV specifically targets marginal metallophilic macrophages (MMMs). Expression of VV transgene in the marginal zone of the spleen^13^ and detection of viral antigens in macrophage-like cells^14^ support the hypothesis. Moreover, non-attenuated VV strain infects splenic MMMs and subcapsular sinus macrophages (SSMs) of the lymph nodes at 4–24 h after systemic administration.^57^^,^^58^ Even with intranasal injection, virus replication can be detected in the spleen and lymph nodes,^4^ suggesting that these organs are highly permissive to the infection.

Interestingly, another member of oncolytic poxviruses—myxoma virus (MYXV) —transiently infects liver macrophages (Kupffer cells) in mice.^22^ MYXV is a rabbit-specific virus,^59^^,^^60^ and its oncolytic activity in other species is associated with hyperactivation of the serine/threonine kinase Akt in transformed cells.^61^ It is quite surprising that the virus manages to infect murine Kupffer cells, given that macrophages are extensively armed to fight the pathogens. In particular, when macrophages are exposed to MYXV *in vitro*, the cells rapidly produce tumor necrosis factor alpha (TNF-α) and type I IFN via an RIG-I-dependent mechanism limiting viral replication.^62^ Further studies are needed to elucidate the mechanisms of macrophage permissiveness for oncolytic poxviruses and to evaluate the clinical importance of the infection.

### Herpes simplex virus

The only OV currently approved by the US Food and Drug Administration (FDA), Talimogene laherparepvec (T-VEC; Imlygic), belongs to the *Herpesviridae* family. Several other oncolytic strains of herpes simplex virus (HSV) are currently under investigation. While i.t. injection is the most common administration route for HSV, virus delivery through the vasculature has also shown promise.^63^, ^64^, ^65^ Most of the HSV-based vectors contain deletions in the neurovirulence gene *ICP34.5* that limit virus replication to Ras-overexpressing tumor cells. Another mechanism of HSV selectivity relies on the inactivation of *ICP6* gene encoding a viral homolog of the cellular ribonucleotide reductase (RR). This enzyme is essential for the production of deoxyribonucleotides, and the replication of the mutant virus is thought to be restricted to actively dividing cancer cells with high levels of RR.^66^ Meanwhile, several research groups provide evidence that oncolytic HSV infects ECs in the TME (Table 1).

Infection of tumor endothelium, but not normal endothelium, was shown both after i.t. and i.v. administration of HSV.^23^^,^^24^ The infection was confirmed by detection of viral proteins,^23^^,^^25^ DNA,^23^ and capsids^24^ in tumor ECs. Interestingly, murine and human ECs have intrinsic susceptibility to HSV, as *in vitro* they are more sensitive to the replicative and cytotoxic effects of oncolytic HSV than other normal cell cultures.^23^^,^^24^ HSV production in HUVECs can be further increased by adding recombinant VEGF, one of the key regulators of tumor neoangiogenesis.^11^ Consistent with this observation, ECs isolated from murine and human cancers are susceptible to HSV infection and killing *ex vivo* while endothelium from normal organs is refractory.^23^ Such selectivity is thought to be dependent on the high proliferation rate of tumor ECs. Cinatl et al. have shown that the elevated levels of cellular RR are responsible for HUVECs susceptibility to HSV, whereas the Ras pathway does not play an essential role for virus replication in ECs.^24^ Moreover, VEGF activates the expression of herpes virus entry mediator (HVEM) in HUVECs.^23^

The dynamics of HSV infection in ECs have not been studied in detail. Although the virus is detected in endothelium both at early (24–48 h ^24^) and late (7–14 days^23^^,^^25^) time points after treatment, so far there is no evidence for HSV spread to adjacent cells of TME. The role of HSV endothelial infection in cancer therapy is controversial. While some authors report a decrease in the number of tumor-supporting vessels upon virus administration,^23^, ^24^, ^25^^,^^67^, ^68^, ^69^, ^70^ others demonstrate the opposite, angiogenic, effect of HSV.^71^, ^72^, ^73^ The ability of the virus to inhibit angiogenesis was demonstrated in multiple cancers (mouse models of ovarian^23^ and squamous cell^70^ carcinomas; human xenograft models of rhabdomyosarcoma,^24^ glioma,^67^^,^^69^ and malignant peripheral nerve sheath tumors^25^) as well as in Matrigel plug assay *in vivo*.^24^^,^^70^ The observed antiangiogenic effect is presumably mediated by upregulation of tissue factor on infected ECs and subsequent prothrombotic changes in tumor vasculature.^23^

On the other hand, HSV-induced angiogenesis was documented in xenograft models of human glioma^71^^,^^72^ and, more importantly, in patients with recurrent breast cancer.^73^ The increase in mean vessel diameter at later time points is associated with upregulation of angiogenic factor CYR61^71^ and reduced levels of antiangiogenic thrombospondins (TSP-1 and TSP-2^72^). The authors hypothesize that while, early after infection, HSV inhibits angiogenesis by direct targeting of ECs, later the rebound effect occurs due to upregulation of angiogenic pathways, such as VEGF induction by CpG motifs in viral DNA.^71^^,^^73^ Another possible explanation for the conflicting results was provided by Huszthy et al.^68^ In that study, the antiangiogenic effect in the focus of HSV infection was accompanied by elevated blood vessel counts in non-infected areas of the tumor. Collectively, these results indicate that both temporal and spatial dynamics of infection should be taken into consideration to comprehensively evaluate the impact of HSV on tumor ECs.

### Reovirus

Reovirus type 3 (Dearing strain) is naturally oncolytic and does not require molecular engineering to target cancer cells. Increased susceptibility of transformed cells to reovirus is mostly attributed to overexpression of EGFR, which activates the Ras signaling pathway. Activated Ras inhibits protein kinase receptor (PKR) activity through a variety of mechanisms, thereby preventing PKR-mediated shutdown of host protein synthesis. Moreover, in normal cells, reovirus uncoating during cell entry is restricted by low cathepsin B and L levels, while, in Ras-transformed cells, overexpression of the cathepsins enhances the efficiency of reovirus uncoating.^74^ So far, in clinical trials, reovirus has been administered via the systemic route more often than any other OV.^1^ In this regard, the ability of the virus to infect vasculature and leukocytes is of special interest (Table 1).

EC infection was shown in three out of nine patients with brain tumors after systemic injection of reovirus.^26^ The early studies demonstrated that ECs deprived of and then re-exposed to a VEGF burst became permissive for reoviral replication and oncolysis.^75^ The induction of the proviral state in HUVECs is established through VEGFR2, while activation of natural killer (NK) cells by reovirus-exposed ECs is predominantly VEGFR1 dependent.^76^ The authors conclude that VEGFR2 intracellular signaling enhances reovirus replication in ECs, similar to that in Ras-transformed tumor cells, and activation of innate immune cells through VEGFR1 could be responsible for the vascular collapse. In agreement with these *in vitro* findings, pretreatment of animals with VEGF before reovirus administration leads to tumor vessel disintegration and a decrease in TIE2 endothelial marker expression.^75^ Of note, in immunodeficient severe combined immunodeficiency (SCID)/non-obese diabetic (NOD) mice, i.t. administration of oncolytic reovirus results in disseminated infection of ECs in normal vessels and development of vasculitis in feet, tails, distal legs, and ears.^27^ Additionally, in these mice, virus infects the myocardium and skeletal muscles. In line with this finding, the immunocompetent rats have the highest reovirus titers in the heart at 72 hpi.^28^ Although clinical signs of vasculitis and myocarditis are revealed only in mice with severe immunodeficiency, it cannot be excluded that in immunosuppressed patients reovirus may yield similar pathology. Indeed, cardiotoxicity upon i.v. injection of reovirus was reported in immunocompetent mice treated with cyclophosphamide, an effect that was attributed to the ablation of humoral antiviral response.^77^

The ability of reovirus to bind to peripheral blood leukocytes is shown both in mice^78^ and humans.^26^^,^^79^, ^80^, ^81^, ^82^ There is a broad range of cells that bind reovirus in the blood flow, including dendritic cells (DCs),^83^^,^^84^ monocytes,^26^^,^^78^^,^^80^^,^^81^ myeloid-derived suppressor cells (MDSCs),^85^ granulocytes,^26^^,^^82^ T cells,^84^ B cells,^26^ and NK/NK T cells,^26^ and some of these interactions may potentially lead to the infection. For instance, reovirus can productively infect murine monocytes,^78^ although the finding is not confirmed in human peripheral blood mononuclear cells.^80^^,^^81^ Likewise, murine,^83^ but not human,^84^^,^^86^ DCs are able to support virus replication *in vitro*, suggesting that species specificity could determine the efficiency of infection. The molecular mechanisms that make some leukocytes, but not others, sensitive to the virus are poorly understood. It has been shown that the expression of reovirus-specific JAM-1 receptor is not required for virus binding to DCs, while sialic acid, which represents an alternative cellular target for reovirus, is important for effective virus entry.^84^ For monocytes, the virus internalization is enhanced in the presence of neutralizing antibodies (NAbs), with FcγRIII playing a distinct role in the uptake of the reovirus-NAb complexes.^78^^,^^80^

Thus far, reovirus replication in immune cells has been documented only *in vitro* and the role of this infection is mostly regarded in the context of cell-based virus delivery. It is not clear if leukocytes can be infected by reovirus *in vivo*, but there is indirect evidence supporting this possibility. Similar to dynamics of splenic infection described for other OVs, a transient increase in viral load was detected in the spleen of rats within 24 h upon reovirus systemic injection.^28^ Moreover, reovirus can be detected in the spleen of mice following i.t. injection.^87^ These findings implicate that splenocytes are permissive for reovirus infection, although the exact target cell has not been identified yet.

Reoviruses typically spread via the respiratory or enteric routes.^88^ Although the infections are usually mild or subclinical, this natural tropism should be considered in the context of OVT safety. The reoviruses differ in their capacity to replicate at mucosal sites. While reovirus strain type 1 replicates to high titers and spreads systemically, strain type 3 (used for OVT) does not.^89^ Consistent with these findings, there are no reports on respiratory or intestinal tract side effects following oncolytic reovirus infusion in humans^90^ or rodents.^28^^,^^75^

### Rhabdoviruses

The lack of pre-existing human immunity against oncolytic rhabdoviruses makes them an attractive candidate for systemic cancer therapy. Vesicular stomatitis virus (VSV) and Maraba virus are currently under investigation in several clinical trials.^91^ Oncoselectivity of rhabdoviruses is based on the lower type I IFN-associated antiviral potential of cancer cells compared with normal cells.^91^ To further improve the safety of the therapy, attenuated strains (VSV^Δ51^ and Maraba MG1) were generated with the defects in genes responsible for inhibition of antiviral immunity.^92^^,^^93^ Another commonly used VSV strain (VSV-IFNβ) carries the *IFNβ* transgene, which further activates an innate antiviral response in normal cells.^94^ Nevertheless, attenuated rhabdoviruses are still able to infect various non-tumor targets (Table 1).

Infected ECs were found in murine CT26 tumors, but not in healthy tissues within 24 h after VSV i.v. injection.^29^ The preferred susceptibility of tumor vessels to VSV is most likely attributed to VEGF overproduction, as the virus demonstrates enhanced infectivity in HUVECs pretreated with tumor-conditioned media or recombinant VEGF.^11^ Infection of ECs coincides with a dramatic decrease in tumor perfusion both in murine tumor model^29^ and human xenografts.^33^ Interestingly, vascular collapse and subsequent tumor necrosis is a neutrophil-dependent phenomenon. OV infection within tumors triggers the release of cytokines (CXCL1 and CXCL5) that attract neutrophils, and these cells, in turn, mediate intratumoral clot formation.^29^^,^^33^ The authors speculate that neutrophil recruitment and vascular shutdown are dependent on endothelial infection; however, it cannot be excluded that neutrophils respond to the infection of tumor cells rather than endothelium.

Similar to ECs, fibroblasts isolated from tumors or pretreated with tumor-conditioned media are more sensitive to VSV and MG1 infection compared with normal fibroblasts. This effect is mediated by tumor growth factor β1 (TGF-β1), which suppresses the transcription of antiviral response genes in cancer-associated fibroblasts (CAFs) and renders them sensitive to virus infection.^95^ This well-designed study provides evidence for CAF infection *in vitro* but does not explore the possibility of targeting CAFs following systemic VSV administration.

Biodistribution studies in mice indicate that VSV transiently replicates in the spleen^30^^,^^33^ and lymph nodes,^30^ reaching a peak of infection at 8–24 h after i.v. injection.^33^^,^^34^ Similar dynamics of MG1 titers in murine spleen have been reported.^36^ Specific targeting of secondary lymphoid organs by VSV and MG1 is also reproduced in other species: beagle dogs^35^ and macaques,^37^ respectively.

There are several targets for VSV in secondary lymphoid tissues. MMMs capture VSV immediately after i.v. injection^30^ and express viral transgene as early as 8 hpi.^30^^,^^31^ Simultaneously, infection of SSMs is observed in the lymph nodes.^30^^,^^31^ The mechanism of macrophage permissiveness to the attenuated virus is unknown, although studies on wild-type (wt) VSV provide some clues. First, the overall accumulation of wt VSV in the spleen is mediated by natural immunoglobulin (Ig) M antibodies^96^ and specific targeting of MMMs is complement dependent.^97^ Second, opposite to the red pulp macrophages in the spleen and Kupffer cells in the liver, MMMs upregulate Usp18, an inhibitor of type I IFN signaling pathway. As a result, these cells have an impaired antiviral response and allow anatomically restricted replication in the splenic marginal zone.^98^ Similarly, SSMs of the lymph nodes capture wt VSV^99^ and allow transient replication upon subcutaneous injection.^57^^,^^100^ Virus retention in SSMs requires neither complement nor natural antibodies. Scavenger receptor has been proposed as a mediator of virus binding to these cells,^99^ but the hypothesis has not been tested yet. Recently, TLR7 has been identified as a major factor that promotes controlled replication of wt VSV in SSMs.^101^

Notably, lymph node infection develops within hours after VSV systemic injection, suggesting that the virus reaches SSMs almost immediately after treatment. Rapid trafficking of virions to the draining lymph node is typical for subcutaneous administration^99^^,^^101^ but quite surprising for systemically injected VSV. Possibly, virus delivery to SSMs is mediated by cell carriers that enter the lymph node through the blood vessels rather than passive lymph drainage. T cells loaded *ex vivo* with VSV are able to deliver the virus to the lymph nodes upon adoptive transfer.^102^ Another potential candidate for virus trafficking to the lymph node is a B cell, which binds VSV immediately after injection.^30^ Indeed, B cells have been shown to deliver VSV to follicular regions in the spleen.^103^ Moreover, the expression of the viral transgene is detected in splenic B cells as early as 6–8 h following the systemic injection.^30^^,^^32^ DC is another target for rhabdoviruses, as shown by both *in vitro*^103^^,^^104^ and *in vivo* studies.^30^ Rhabdovirus infection of secondary lymphoid tissues could be important for boosting antitumor immune responses^36^; however, experimental proof of this exciting hypothesis remains a work in progress.

### Measles virus

Edmonston strain of measles virus (MV-Edm) selectively targets tumor cells through CD46, which is known to be overexpressed in many human malignancies. For clinical trials, the virus was modified by incorporating a sodium iodide transporter (NIS) to monitor the infection through administration of iodine-123 via single-photon emission computed tomography (SPECT) imaging.^105^

There are conflicting data on the ability of MV to target tumor ECs. Using the virus retargeted against the urokinase receptor (MV-Edm-uPAR) for enhanced binding to cancer cells, Jing et al. revealed the infection of tumor ECs after i.t. and i.v. administration. The sensitivity of tumor endothelium was attributed to the overexpression of uPAR in ECs as *in vitro* tumor-conditioned media or VEGF treatment upregulated uPAR expression in HUVECs.^38^ In contrast, Ong et al. have reported that non-targeted MV-Edm infects perivascular cells that are negative for endothelial marker CD31 and have a circular shape, and only arginine-glycine-aspartic acid (RGD)-displaying viral constructs are able to target ECs.^106^ Opposite to humans, in mice, the expression of MV-Edm entry receptor CD46 is mainly restricted to the testes, and therefore more relevant preclinical models are needed to fully map the cells infected in the TME.

In humans, monocytes and macrophages represent a primary target in natural MV infections and are readily transduced by MV-Edm *in vitro*.^107^ Using measles-susceptible transgenic mice, Myers et al. have shown the increased virus titers in the spleen compared with other organs on days 2 and 22 after systemic MV-Edm injection.^39^ In agreement with these data, splenic MMM is identified as the preferred virus-infected host cell at 48 h after i.v. administration of MV-Edm.^40^ Moreover, intraperitoneal injection of MV-Edm also results in viral transgene expression in MMMs,^40^ whereas in the lymph nodes viral RNA is found in SSMs following intranasal administration.^108^ These results demonstrate that a relatively low number of viral particles leaking from the site of injection is sufficient to infect MMMs and SSMs. To uncover the mechanism responsible for MV-Edm sequestration in the spleen and liver, biodistribution studies were performed in CD46− athymic and CD46+ transgenic mice. While the absence of CD46 did not affect MV-Edm accumulation, the blockage of scavenger receptor significantly reduced virus uptake by macrophages.^109^ The subpopulations of MV-capturing splenocytes have not been identified in this study and thus it is not clear if scavenger receptor is responsible for MV binding to MMMs or other macrophages.

### Adenoviruses

Adenoviruses (Ads) are extensively used as vaccine platforms, vehicles for gene delivery, and oncolytic agents. Among other OVs, Ad is unique in its ability to transduce hepatocytes *in vivo*, and multiple strategies have been suggested to limit the off-target infection. The replication-competent Ad5-Δ24-RGD has an inactivated tumor suppressor retinoblastoma protein (pRb)-binding function of E1A (the delta-24 modification) and RGD motif-containing integrin-targeting peptide (RGD-4C) inserted in the HI loop of the fiber knob domain for coxsackievirus and Ad receptor (CAR)-independent infection of cells by targeting mainly αVβ3 and αVβ5 integrins.^110^ In Ad5-Δ24-RGD-injected mice, an intense diffuse viral E1A expression in the liver was demonstrated, and the Е1АΔ24 modification failed to prevent hepatotoxicity.^41^ However, the liver transduction was significantly reduced when E1A promoter was replaced by the human E2F1 promoter preceded by a synthetic polyA signal and additionally insulated by a fragment from the myotonic dystrophy locus with insulator activity.^41^ The viral *E1* gene expression driven by telomerase reverse transcriptase-specific promoter did not prevent expression of the viral *E1A*, *E2*, and *E4* genes in the liver.^43^ Likewise, the application of neuroendocrine-selective CgA promoter to control the viral *E1A* expression was not sufficient to abrogate luciferase (Luc) activity in the liver of mice receiving an i.v. injection of *E1B*-deleted Ad5-CgA-Fluc reporter virus with *E1A* substituted by *Luc*. Notably, the additional post-transcriptional control of Luc expression by six tandem repeats of the target sequence for the liver-specific miR122 completely blocked Luc activity and prevented hepatotoxicity in mice injected with Ad5-CgA-E1A-miR122.^42^ Enadenotucirev, a vector derived from a pool of Ad serotypes (subgroups B–F), represents Ad11p with a nearly complete E3 region deletion, a smaller deletion in the E4 region, and a chimeric Ad3/Ad11p E2B region.^111^ Ad11p infects cells by binding to CD46 and desmoglein 2 (DSG2).^112^ Although the murine cells lack CD46 and do not support efficient transduction by DSG2-dependent human Ads,^112^^,^^113^ the reporter replication-competent Ad11p is still capable of transiently infecting liver cells in mice^44^ as measured by Luc activity under cytomegalovirus (CMV) promoter regulation. Meanwhile, no transgene signal could be detected in the liver by linking the Luc expression to the activity of the virus major late promoter (MLP), suggesting a lack of active replication and reproduction.^44^ At the same time, *in vitro* hepatocytes support higher replication levels of Enadenotucirev compared with other human normal primary cells, pointing to intrinsic sensitivity of hepatocytes to the virus.^114^

Although the exact mechanism responsible for Ad tropism to hepatocytes is still debatable (reviewed in Stepanenko and Chekhonin^115^), the susceptibility of the cells to viral infection is most likely associated with high expression levels of the virus entry receptors, rather than impaired IFN response. Ad5 infects the murine liver in a CAR-, integrin-, fiber shaft KKTK motif-, and hepatic heparan sulfate glycosaminoglycan (HSG)-independent, but fiber-, hexon-, and blood factor X (FX)-dependent manner.^115^ It has been suggested that FX also protects virions from attack by natural antibodies, thus increasing the number of viral particles available for binding to the host cells.^116^ However, inhibition of FX not only limits liver infection but also increases the number of infected cells in other organs.^117^ These results indicate that FX is more important for hepatocyte targeting^118^ than for retaining Ad infectivity in the blood flow. Another factor potentially contributing to hepatocyte infection is an extremely high virus to cell ratio in the liver compared with other tissues, since more than 90% of the systemically injected dose is sequestered in the liver.^115^^,^^119^

Significant efforts have been applied to deliver genes to tumor endothelium using transcriptionally targeted Ads. These strategies have been discussed in detail previously^120^ and are largely out of the scope of the current review, which focuses on the intrinsic capacities of OVs to infect non-tumor cells. To the best of our knowledge, infection of tumor ECs has not been reported for oncolytic Ads undergoing clinical trials. However, biodistribution studies of Ad constructs that are currently tested in preclinical models demonstrate the capacity of Ad to selectively infect tumor endothelium.

*In vitro*, VEGF-stimulated HUVECs upregulate the expression of Ad coreceptor integrin αVβ3. Consistently, Ad retargeting from CAR to αVβ3 results in twice as efficient HUVEC infection. Of note, transcriptional targeting by using endothelial receptor promoter Tie2 does not further increase the transduction. The authors conclude that the expression of a reporter transgene in ECs is limited more by receptor availability than by the activity of the transcriptional promoter. Importantly, systemic injection of Ad5-RGD targeting αVβ3 due to an insertion of RGD-4C sequence in the HI loop of the fiber protein leads to infection of ECs in tumor vessels, but not normal vessels.^121^

In HUVECs and human aortic ECs (HAECs), the fiber-chimeric Ad5/35 virus with the serotype 5 fiber knob and shaft domains replaced with those from serotype 35 results in between 1 and 3 orders of magnitude higher gene expression than parental Ad5. This striking difference is likely attributed to high expression levels of CD46, a well-known receptor for Ad35, in ECs. Accordingly, upon intrahepatic infusion, Ad5/35 infects ECs in the hepatocarcinoma rim, whereas Ad5 is found mainly in liver parenchyma.^122^

A comparative study of EC infection by Ad5 expressing EGFP under either the CMV promoter (Ad5CMV) or endothelial-specific enhancer/promoter roundabout4 (Ad5ROBO4)^117^ provides intriguing results. Systemically administered Ad5CMV infects ECs in tumors and multiple organs in immunodeficient mice expressing human CAR, but not in CAR non-transgenic animals. Similarly, ubiquitous CAR expression dramatically increases endothelial infection by Ad5ROBO4 both in tumors and normal tissues. Of note, enhanced targeting of tumor endothelium by Ad5ROBO4 is achieved in CAR non-transgenic mice pretreated with warfarin that inhibits FX-mediated virus uptake by the liver. These data suggest that Ad5 utilizes both CAR-dependent and CAR-independent mechanisms to bind ECs and that FX is not essential for entering tumor ECs as opposed to hepatocytes.^118^ The same study reports the infection of circumferential microvessels immediately adjacent to ovarian follicles, but not in stromal microvessels of the ovaries. Conceivably, the off-target infection is due to a high proliferation level of ECs in perifollicular vessels.^117^

A recent study^45^ demonstrates infection and depletion of fibroblast activation protein α–positive (FAP+) cells in murine glioma upon i.t. injection of the replication-competent Ad5 ICOVIR15, which expresses the viral *E1AΔ24* under regulation of a modified endogenous E1A promoter, containing eight E2F-1–binding sites and one Sp1-binding site.^123^ *Ex vivo* infection of freshly isolated FAP+ cells from mouse glioma confirmed the susceptibility of stromal FAP+ cells to Ad5.^45^ As both fibroblasts and pericytes express FAP, more detailed phenotyping is needed to identify the infected cells. Notably, several independent studies provide indirect evidence for tumor pericyte sensitivity to Ads. Thus, i.v. administration of Ad5 into human CAR-expressing mice results in infection of both tumor ECs and perivascular stromal cells, while the virus fails to infect any cells in CAR non-transgenic mice.^117^ Consistent with this finding, virus retargeting from CAR to αVβ3 is accompanied by a 2-fold decrease in transgene expression in pericyte-like C3H/10T1/2 cells. Moreover, Ad modified for enhanced targeting of neovasculature infects not only ECs but also pericytes identified as CD13+ perivascular mesenchymal cells.^121^ Finally, Ad vector designed for targeting tumor endothelium has been shown to infect cells that express CD31 and platelet-derived growth factor receptor β (PDGFRβ), a well-described pericyte marker.^124^

Among other organs, the spleen accumulates the highest viral titers after systemic injection of murine AdMAV-1 dlE102, an analog of human ICOVIR-5.^46^ Infection of splenocytes is detectable by 72 h after injection of Ad5, being more pronounced in mice expressing human CAR as well as in CAR non-transgenic mice pretreated with warfarin. These results suggest that the virus entry in splenocytes is at least partially CAR dependent and that liver detargeting by depletion of FX enhances infection in the spleen.^117^ In patients receiving i.t. injection of Enadenotucirev, hexon staining was found in tumor-draining lymph nodes, although the identity of the virus-positive cells was not established.^47^ While it is not clear yet which cells are sensitive to oncolytic Ads in secondary lymphoid tissues, the cellular targets for non-attenuated Ads in the spleen and lymph nodes are well defined. Systemically injected Ad5 accumulates in the marginal zone of the spleen,^119^^,^^125^, ^126^, ^127^ and lymph-borne Ad5 is entrapped by SSMs.^99^ Moreover, the E1-deleted Ad5-LacZ reporter virus transduces MMMs and splenic DCs as early as 6 hpi.^125^ The specific targeting of SSMs/MMMs could be attributed to their anatomical location and/or expression of Ad-binding scavenger receptor SR-A6 (MARCO),^128^ on the surface of the macrophages.

### Picornaviruses

Picornaviruses represent a heterogeneous group of viruses, with many of them showing encouraging antitumor efficacy in patients. While poliovirus RIPO (PVSRIPO) and echovirus 7 (Rigvir) are mostly tested for local administration, coxsackievirus A21 (CVA21, CAVATAK) and Seneca Valley virus (SVV, NTX-010) can be delivered systemically.^129^ The tumor selectivity of picornaviruses arises from the overexpression of the virus entry receptors on malignant cells: intercellular adhesion molecule-1 and/or decay-accelerating factor (CVA21),^130^ anthrax toxin receptor 1 and sialic acids (SVV),^131^ CD155 (PVSRIPO),^132^ and integrin α1β2 (echoviruses).^133^

Oncolytic picornaviruses are generally well tolerated in clinical trials and the off-target infection has not been reported thus far. However, *in vitro* PVSRIPO infects macrophages and DCs, inducing the expression of major histocompatibility complex class II and production of IFN-β and IL-12.^134^ Importantly, the virus neither kills the cells nor interferes with their ability to engage T cells. These stimulating properties could be an important component of the therapy if PVSRIPO is also able to infect the cells *in vivo*. The permissiveness of antigen-presenting cells for the virus is likely attributed to a high expression level of CD155 on macrophages and DCs.^135^ Of note, CD155 is also upregulated in tumor-associated vasculature.^136^ Similarly, SVV entry receptor is overexpressed not only on malignant cells but also on tumor endothelium, pericytes, and CAFs.^137^ Currently, there is insufficient information regarding picornaviruses biodistribution on cellular level, and further studies are required to show if these viruses share the same non-tumor targets with other classes of OVs.

## Is an infection of non-cancer cells important for oncolytic virotherapy?

### Infection in the tumor microenvironment

**Figure 1:**
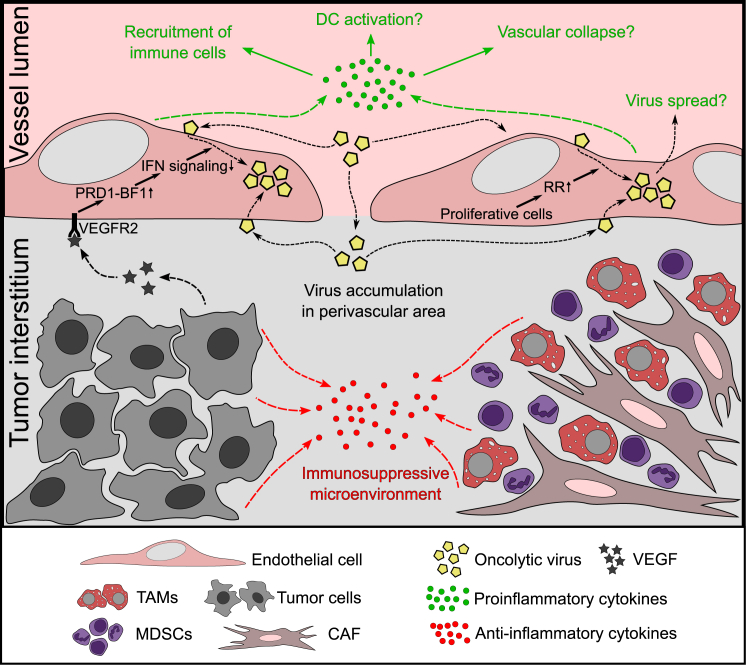
Tumor microenvironment sensitizes endothelium for OV infection Upregulation of proangiogenic signaling in a growing tumor makes ECs vulnerable to OV infection via several mechanisms: (1) increased permeability of neovessels facilitates OV accumulation in the perivascular area; (2) actively proliferating ECs provide OVs with ribonucleotide reductase (RR), the enzyme essential for virus replication; and (3) vascular endothelial growth factor (VEGF) binding to VEGFR2 activates transcription repressor PRD1-BF1, which interferes with genes involved in type I interferon (IFN)-mediated antiviral signaling. Additionally, cancer cells and reprogrammed stromal cells create an immunosuppressive milieu favoring OV replication in the tumor microenvironment. In turn, EC infection may promote antitumor responses by (1) secreting proinflammatory cytokines; (2) recruiting/activating immune cells; (3) launching vascular shutdown; and (4) supporting OV spread in the tumor. TAMs, tumor-associated macrophages; CAF, cancer-associated fibroblast; MDSC, myeloid-derived suppressor cell.

Among non-malignant cells of the TME, EC is the most frequently detected target for various systemically delivered OVs, including VV, HSV, reovirus, VSV, and Ad (Table 1). The promiscuous EC infection by the viruses belonging to different families suggests that this phenomenon is attributed to the biology of tumor ECs rather than viral properties. Tumor vessels are distinct from normal vasculature in several ways as a result of chronic exposure to cytokines and growth factors secreted by cancer cells, such as VEGF, EGF, PDGF, TGF-β1, TGF-β2, fibroblast growth factor 1 and 2, IL-6, IL-8, and IL-10.^95^^,^^120^^,^^138^ Several studies point to VEGF as a major determinant of EC permissiveness to OVs. Indeed, pretreatment of HUVECs with VEGF sensitizes the cells to VV,^10^, ^11^, ^12^ VSV,^11^ Maraba,^11^ HSV,^11^ reovirus,^75^^,^^76^ and MV.^38^ Moreover, the importance of VEGF for tumor endothelial infection was proved by using blocking antibodies to VEGF/VEGFR2 in VV-treated mice.^11^ VEGFR2 signaling is responsible for the suppression of type I IFN-mediated antiviral control leading to EC infection.^11^ Additionally, to create a tumor-supportive milieu, cancer cells recruit and reprogram leukocytes and secrete soluble immunosuppressive cytokines (e.g., TGF-β, IL-10),^138^ which may also favor viral replication in non-cancer cells. High proliferative and metabolic activity of ECs exposed to angiogenic factors is another possible explanation for their vulnerability to OVs.^76^ Finally, tumor neovessels are characterized by enhanced permeability,^139^ which facilitates virus retention in the perivascular area (Figure 1).

Compared with other cells of the TME, ECs are most exposed to blood-borne viruses and, intuitively, the direct access of endothelium to circulating viruses should increase the chances of infection. However, such a simplistic approach could be misleading as it is not clear if OVs reach EC from the luminal side or the virions leak into the tissues and enter the cell from the basolateral side.^106^ Revealing the OV entry side is closely related to the identification of the receptors that mediate viral uptake.

Infection by a wide range of OVs argues against the overexpression of a virus-specific receptor on tumor ECs as the viruses utilize different strategies for entering cells. For instance, VSV infection requires LDL receptors, while MV binds the cell through CD46. It is more likely that endothelium-virus interaction is mediated by pattern recognition receptors (PPRs) that bind pathogen-associated molecular patterns, or by capturing opsonized virions through Fc or complement receptors. Although the role of these receptors in EC infection has not been studied in detail, there is a report on increased EC infection by reovirus in SCID/NOD mice lacking antibodies.^27^ These results suggest that immunoglobulins and Fc receptors are dispensable for EC-virus interactions; however, the results obtained in mice with severe immunodeficiency should be interpreted with caution. In contrast, scavenger receptors (a subclass of PPR) have been shown to mediate uptake of Ad,^128^ HSV,^140^ VV,^141^ and MV^109^ by various host cells. Whether endothelial scavenger receptors^142^ mediate OV infection is a matter for future investigation.

The importance of EC infection for OVT is debatable (Figure 1). So far there is no direct evidence that ECs can serve as a source of the second wave of infection contributing to the spread of the virus in the tumor. The fact that tumor EC infection by OVs is not consistently found by different research groups^17^^,^^19^^,^^51^^,^^52^^,^^143^^,^^144^ suggests that the number of infected cells is low and the infection is most likely abortive. In this case, using viral constructs with fluorescent transgenes under the control of early versus late viral promoters could yield conflicting results. It would be important to reveal to what extent the virus life cycle is processed in ECs; in particular, if the viral genes are expressed to the levels that trigger cell-autonomous or immune-mediated cell death.

Multiple studies have linked EC infection with the antiangiogenic effect of OVT. Indeed, a decrease in the vessel density is one of the most remarkable histological findings upon systemic or local OV administration. The antiangiogenic effect is an important component of cancer therapy as it deprives growing tumors of blood supply^33^ and prevents metastasis.^145^ The ability to destroy lymphatic vessels has the advantage of blocking another important route of cancer dissemination.^52^ In this context, it is tempting to speculate that vascular collapse is driven by infected ECs, but there are some missing links in this hypothesis. First of all, we still do not know whether ECs survive the infection or undergo some type of cell death. An inflammatory response is another potential mechanism of OV antiangiogenic activity that could be tied with EC infection. For instance, VV^33^^,^^50^ and VSV^29^^,^^33^ systemic administration leads to massive infiltration of neutrophils triggering clot formation and loss of perfusion in the tumor. However, it is not clear if neutrophils respond to infected ECs or infected tumor cells. The importance of EC infection for triggering vascular collapse is compromised by studies where decreased tumor perfusion is observed in the absence of VV infection of ECs.^17^^,^^19^^,^^52^ Moreover, vascular shutdown occurs after treatment with Sindbis virus^139^ and Newcastle disease virus (NDV),^146^ OVs that have never been reported to target endothelium.

In the light of these findings, the assumption that vessel disruption is mediated by downregulation of angiogenic factors (VEGF, hypoxia-inducible factor-1) in infected tumor areas^53^^,^^147^^,^^148^ seems to be better grounded than the hypothesis of vascular collapse triggered by EC infection. Of note, Enadenotucirev, the virus that fails to infect CD46-negative murine ECs, downregulates VEGF in infected areas of human tumor xenografts, which coincides with the decreased vascular perfusion.^147^ Moreover, Ad infection of cancer cells stimulates immune cells to produce IFN-γ, a potent antiangiogenic factor. Consistently, the antiangiogenic effect of Ad is less apparent in SCID mice lacking immune responses and in immunocompetent mice following neutralization of IFN-γ.^149^

Finally, it should be noted that the concept of OV-mediated vascular collapse is also a matter of debate. While some authors report the decrease in the number of tumor vessels in HSV-treated tumors, others provide evidence for angiogenic properties of the virus. Similarly, there is no consensus regarding VV impact on tumor vasculature: along with studies demonstrating virus antiangiogenic effect, there is evidence that VV increases vessel permeability^13^^,^^51^ and even upregulates VEGF.^49^

Focusing immune response to tumors is another potential implication of EC infection. One can suppose that infected endothelium can serve as a beacon for effector immune cells. Bridle et al. have shown that antitumor immune response primed with Ad and boosted with VSV expressing the same tumor antigen is more effective in tumor-bearing mice than in healthy animals.^150^ This effect was attributed to VSV replication in tumor cells. Conceivably, infected ECs could play the same role in boosting antitumor immunity locally in the tumor bed, especially in the situation when cancer cells are resistant to viral oncolysis.

EC involvement in the immune response could be more complicated. The lessons learned from the infection of CD169+ cells (see below) suggest that transient replication of the pathogen in the host cell is crucial for limiting virus spread and eliciting a rapid antiviral response. In this scenario, EC infection may protect neighboring cancer cells from infection by creating a proinflammatory milieu. Further studies are needed to understand if EC acts as an immune sentinel in the TME and to identify the potential benefits and drawbacks of endothelial infection for OVT.

The ability of OVs to infect ECs can be also important for the delivery of therapeutic genes selectively to a tumor milieu. Tremendous efforts have been applied to target tumor endothelium by Ad, AAV, lentiviruses, and retroviruses.^120^^,^^151^, ^152^, ^153^ The intrinsic capacity of OVs to infect tumor ECs broadens the arsenal of potential vectors for cancer gene therapy.

It cannot be excluded that EC is not the only cell type permissive for OVs in the TME. Thus, *in vitro* CAFs are more sensitive to rhabdoviruses and VV than normal fibroblasts.^95^ Tumor pericytes have recently been identified as a target for Ad following i.t. administration.^45^ Of note, brain and lung pericytes are highly sensitive to natural HIV infection.^154^^,^^155^ Looking closely into the immunohistochemistry results identifying the infected cells, one can see that, very often, virus transgenes are expressed from perivascular cells distinct from CD31+ endothelium.^10^^,^^13^^,^^106^^,^^117^^,^^121^^,^^124^^,^^143^ Precise phenotyping of infected stromal cells may provide novel concepts for fighting cancer by targeting distinct components of the TME.

### Infection in secondary lymphoid tissues

**Figure 2:**
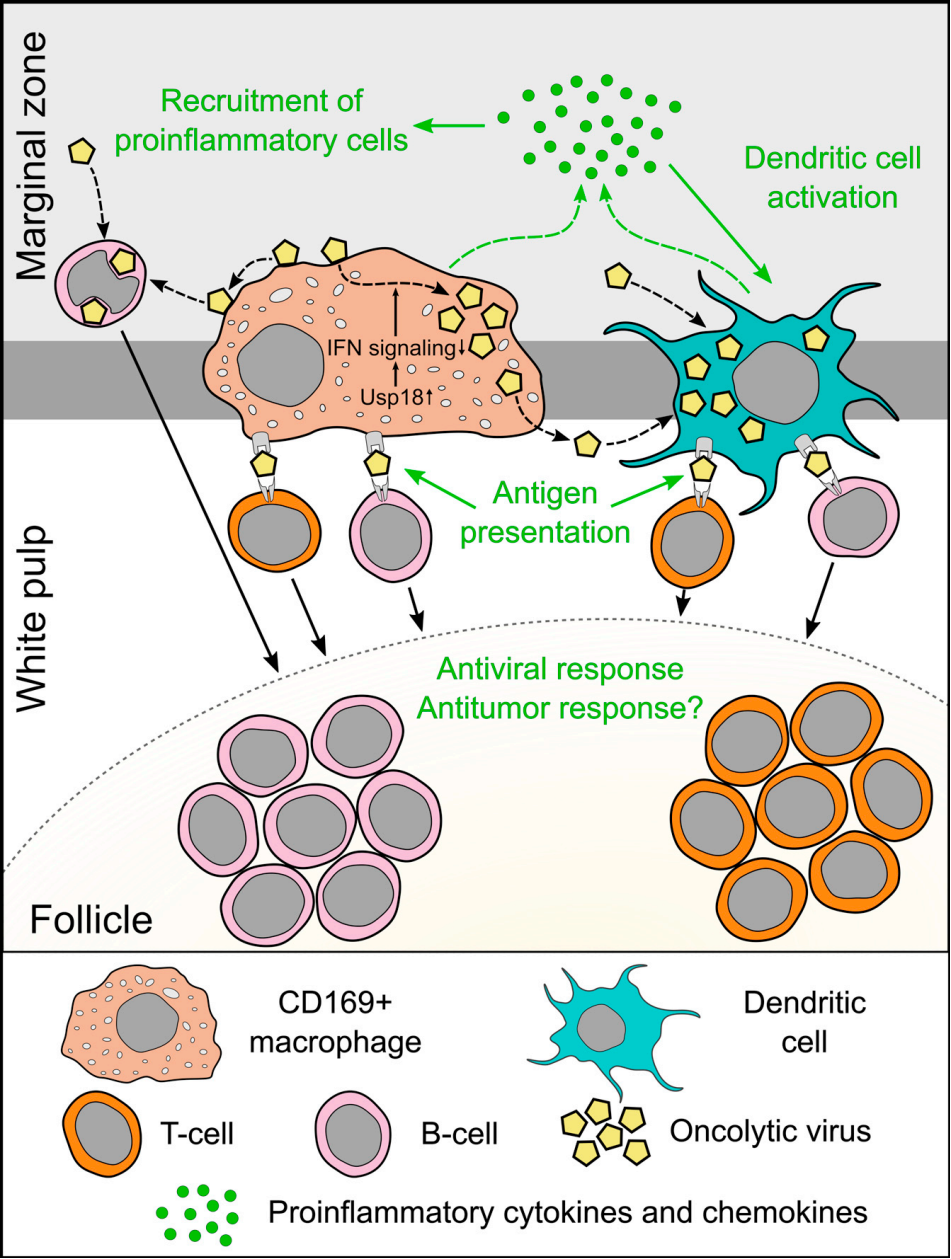
Secondary lymphoid organs allow controlled OV replication (exemplified by spleen) Unique properties of MMMs determine their permissiveness to OVs: due to anatomical location, these cells accommodate high concentration of systemically injected OVs; (2) Usp18 upregulation blocks type I IFN-mediated response in MMMs. Transient viral replication in CD169+ cells is crucial for eliciting rapid antiviral responses by (1) secreting type I IFN and other cytokines; (2) recruiting inflammatory cells; (3) priming T and B cell responses; and (4) multiplying antigen for activation of neighboring dendritic cells. Moreover, MMMs can transfer cell-surface-associated infectious virions to B cells and DCs, contributing to the infection of these cells. Virus replication in DCs further increases type I IFN production in the lymphoid tissues and improves the efficiency of antigen presentation. Additionally, B cells may transfer OVs directly to follicular DCs, enabling boost response in immunized hosts.

Secondary lymphoid tissue is another common target for the majority of OVs (Figure 2). The increased viral titers in the spleen have been shown for VV,^14^, ^15^, ^16^, ^17^, ^18^ VSV,^33^, ^34^, ^35^ MG1,^36^^,^^37^ MV,^39^ Ad,^46^ reovirus,^28^ NDV,^156^^,^^157^ and for the recently described oncolytic alphavirus M1.^158^ Of note, for different viruses, the splenic infection has similar dynamics, reaching a peak within a few hours and gradually decreasing thereafter.^28^^,^^33^^,^^34^^,^^36^^,^^39^^,^^57^^,^^58^

There are several lines of evidence suggesting that CD169+ macrophages (MMMs and SSMs) are the main source of OV replication in the secondary lymphoid organs. First, the transient infection of these cells was directly proved for at least two viruses, VSV^30^^,^^31^ and MV.^40^ Second, in the case of other OVs, the detailed phenotyping of infected cells is lacking, but these cells have macrophage-like morphology and localize in the splenic marginal zone.^13^^,^^14^^,^^117^ Third, the role of MMMs and SSMs in the control of blood-borne^159^ and lymph-borne^160^ infection is well defined.

There is much in common between SSMs and MMMs. These cells are strategically positioned to probe soluble antigens and microbes. The marginal zone of the spleen is a sinusoid system on the border between the red and white pulp, where the blood slows down, allowing for a continuous scan of the antigens by sinus-lining macrophages. Similarly, in the lymph node, SSMs lining the flow of the sinuses directly embrace the lymph. Both macrophage subsets express sialoadhesin (CD169), which binds to α2,3-linked sialic acids on the viral capsid^161^ and exosomes.^162^ It should be noted that, along with CD169+ cells, there is another macrophage subset in the marginal zone of the spleen: marginal zone macrophages. These cells are characterized by high expression levels of the PPR (e.g., C-type lectin SIGN-R1, scavenger receptor A, macrophage receptor with collagenous structure),^159^ potentially contributing to the increased viral sequestration in the proximity to MMMs.

Opposite to other macrophage subsets, CD169+ cells are not extensively armed to clear the viruses despite being abundantly exposed to the incoming pathogens. SSMs are poorly phagocytic and are not able to clear the pathogens directly.^163^ Moreover, MMMs upregulate the expression of IFN inhibitor Usp18.^98^ It is well known that, in mice lacking IFN-α/β receptors, macrophages of multiple organs are the main sites of virus replication.^40^^,^^108^^,^^164^ The constitutive suppression of IFN response in the MMMs combined with high local viral load makes possible the transient replication of a virus (Figure 2).

Why do CD169+ macrophages allow for transient viral replication instead of destroying the pathogens? This question has been extensively studied and the results indicate that the permissiveness of CD169+ macrophages for pathogenic viruses is an elegant evolutionary mechanism of antiviral response. Thus, IFN secretion by VSV-infected SSMs and by plasmacytoid DCs recruited to the sites of infection prevents virus invasion of central nervous system.^100^^,^^165^ Likewise, without the immune protection generated by this layer of macrophages, murine CMV (MCMV) invades deeper into the parenchyma of the lymph node and eventually disseminates to other organs.^166^ Recruitment of NK cells and their IFN-dependent activation by SSMs is another mechanism that can restrict virus dissemination.^167^ The role of the MMM infection in the acute inflammatory response is less studied; however, there are data suggesting these cells have similar functions to SSMs. Thus, MMMs are the major source of IFN production upon systemic injection of UV-inactivated HSV,^168^ and depletion of these cells leads to lymphocytic choriomeningitis virus spread from the spleen to peripheral organs.^169^

Multiplication of viral antigens in secondary lymphoid tissues accompanied by proinflammatory cytokines production is also crucial for the development of adaptive antiviral immunity. In particular, a high virus to cell ratio is needed for DC maturation and activation.^86^^,^^170^ Along these lines, the infection of SSMs stimulates CD8+ T cell recruitment to the periphery of the lymph node, where they form tight interactions with DCs, leading to complete T cell activation within the first 12 h after infection.^57^ Similarly, MMMs expressing Ad-encoding proteins collaborate with splenic DCs for the generation of T cell immunity.^171^ Moreover, VSV replication in MMMs is essential for the induction of adaptive antiviral immunity and survival. Importantly, inactivation of VSV by UV light limits the virus-specific CD8+ T cell response and blunts the induction of neutralizing IgG,^98^ suggesting that viral replication in MMMs is required to control the infection. In summary, CD169+ macrophages not only serve as a “flypaper” for the viruses but actively participate in eliciting inflammatory and immune responses by permitting transient viral replication (Figure 2).

DCs can also be infected by OVs *in vitro*^83^^,^^84^^,^^103^^,^^104^^,^^134^ and in secondary lymphoid tissues of virus-treated mice.^30^ Farrell et al. suggest that, in the lymph node, DCs obtain the virus via infected SSMs as infection of DCs by MCMV is not observed upon the depletion of SSMs.^166^ Indeed, the ability of SSMs to shuttle viral particles across the subcapsular sinus floor to the neighboring cells has been demonstrated.^99^ Of note, Usp18 expression in DCs enhances the presentation of replicating antigen^98^; however, there are no data regarding the role of Usp18 in the capacity of DCs to permit virus infection *in vivo*. A wide range of viruses activate type I IFN production in DCs,^134^^,^^172^^,^^173^ and several reports highlight the role of infected DCs in eliciting immune responses. DCs infected by non-attenuated VV strain are able to present antigen to naive CD8+ T cells in the lymph node.^58^ Likewise, infection of splenic DCs and MMMs by E1-deleted Ad triggers acute inflammation and contributes to the specific cellular immune response to Ad.^125^

While the role of transient infection of CD169+ macrophages and DCs for controlling pathogenic viruses is well documented, its importance for OVT has not been addressed yet. One can suggest that infection of these cells contributes to the development of antiviral immunity and enhanced clearance of OVs, similar to pathogenic viruses. It is also possible that infection of the immune cells promotes antitumor responses by creating a proinflammatory milieu in secondary lymphoid organs. On the other hand, CD169+ macrophages are known to downregulate immune responses to self-tissue,^159^^,^^174^ and their potential role in the development of immune tolerance toward viral and tumor-derived antigens in the course of OVT should be comprehensively evaluated.

Apart from eliminating microbes, SSMs are also capable of capturing lymph-borne tumor cells and prime antitumor CD8+ T cells.^160^ In this regard, it is important to understand how OV replication in CD169+ macrophages influences the capacity of these cells to prevent cancer dissemination. The fate of OV-infected SSMs has not been studied yet, but it is known that non-attenuated viruses disrupt the macrophage layer. A dramatic reduction in the number of SSMs is observed following administration of VV^58^ and MCMV,^166^ which could be either the result of direct viral lysis or IFN-mediated apoptosis. In agreement with these findings, abnormal vacuolized cytosolic compartments and grossly distorted mitochondria are detected in Ad-containing splenic macrophages early (4 hpi) after treatment. The authors demonstrate that the virus-infected cells are rapidly eliminated by neutrophils recruited to the spleen via the chemokine-activating IL-1α-IL-1RI-CXCR2 axis.^119^

Besides the infection in lymphoid organs, there are anecdotal reports of monocyte/macrophage infection by OVs in other tissues, including liver^22^ and tumor.^121^ Due to a wide repertoire of receptors to viral ligands and opsonized pathogens, phagocytes rapidly absorb the major part of systemically administered OVs.^30^^,^^78^^,^^80^^,^^128^^,^^140^^,^^141^ In this regard, evaluation of phagocytic cell infection *in vivo* is challenging as detection of viral genomes or proteins could be either due to phagocytosed or replicating viruses. *Ex vivo* studies demonstrate that monocytes are uniquely susceptible to reovirus,^78^ VSV, VV, and influenza A virus infection, and are able to differentiate into DCs upon exposure to a live virus.^170^ The authors suggested that these cells evolved to support viral gene expression with the likely purpose of enhancing antiviral response by enabling endogenous antigen presentation.^170^

B cells are another target for OVs *in vivo*. Previously we have shown that splenic B cells capture systemically delivered virions immediately after VSV administration and express viral transgene as early as 8 hpi.^30^ This observation is consistent with another report where B cell infection has been found in the splenic follicles.^32^ There are at least two possible explanations for early viral infection in the follicular zone of the spleen. SSMs are able to transfer intact virions to the underlying B cells^99^ and it is tempting to speculate that MMMs in the spleen could have the same function. Alternatively, peripheral B cells or marginal zone B cells might uptake the circulating virus and migrate into B cell follicles. This assumption is supported by the ability of *ex vivo*-loaded B cells to deliver VSV to the follicular regions in secondary lymphoid organs.^103^ Of note, after exposure to the virus *in vitro*, B cells bind VSV but do not allow for viral gene expression.^103^^,^^170^ Nevertheless, both *ex vivo*-loaded and *in vivo*-infected B cells have shown promise for boosting immune response as they can deliver antigen to DCs in follicular regions where DCs cannot be cleared by effector CD8+ T cells providing robust expansion of memory CD8+ T cells.^32^ The ability to infect B cells is not unique to VSV and has been also documented for systemically injected MYXV^175^ and Ed-MV.^176^ Further studies are needed to reveal the mechanism responsible for B cell permissiveness to attenuated OVs.

### Infection in other tissues

Infection of the liver is not typical for clinically used OVs. In this regard, Ad is a remarkable exception as hepatocyte is a primary target for Ad following systemic injection. The virus specificity of the phenomenon suggests that the mechanism of infection is linked to the unique properties of Ad rather than host factors. Virus sequestration in the liver prevents its targeted delivery to tumor sites and may lead to undesired effects. Dose-limiting acute liver toxicity of oncolytic Ads is commonly observed in small animal models,^177^^,^^178^ and, in clinics, transaminitis is among the most frequent side effects of Ad-based OVT.^179^, ^180^, ^181^, ^182^, ^183^

The rapid decrease in liver viral titers and number of transduced hepatocytes indicates that the infection is self-limiting,^42^^,^^44^ but it is not clear if the infected cells survive. The clearance of the cells may occur via direct viral lysis or immune-mediated cell death, and both mechanisms can potentially contribute to toxic effects. It should be noted that the infection of hepatocytes is not the only factor of Ad hepatotoxicity. Virus uptake by liver macrophages leads to a profound release of proinflammatory cytokines and recruitment of immune cells.^184^ This inflammatory response not only helps to control virus spread but also leads to necrotic changes in macrophages and bystander cells.^184^^,^^185^ Although it is problematic to conclude which of these two factors, infection of hepatocytes or excessive host response, contributes more to Ad-related toxicity, the infection may further exacerbate detrimental immune reactions. Regardless of the underlying mechanism of hepatotoxicity, numerous studies demonstrate a correlation between the level of infection and severity of the toxic effects.^41^, ^42^, ^43^^,^^178^^,^^186^ On the other hand, given the established role of the liver in immune surveillance,^187^ it could be also important to study whether OV infection of hepatocytes influences the development of adaptive antiviral and antitumor responses.

Similar to the liver and spleen, lungs sequester a significant part of the systemically injected virions because venous blood flow is first directed through the pulmonary vasculature. Ads transcriptionally targeted for activated ECs in the tumor readily infect the vasculature in the lungs.^117^^,^^188^^,^^189^ Notably, VEGF pretreatment sensitizes lungs to reovirus infection.^75^ Moreover, increased and/or persistent viral titers are detected in the lungs after systemic injection of VV,^17^, ^18^, ^19^^,^^55^ VSV,^33^ NDV,^156^^,^^157^ MV,^190^ and Semliki Forest virus,^191^^,^^192^ and even after local VV administration into the flank tumors.^193^ These data support the possibility of OV replication in the lungs, and further studies are required to reveal cellular targets for the viruses.

Oncolytic VV strains are known to infect skin and mucosa, causing pox-like lesions. A papulopustular rash has been documented both in animal studies^15^^,^^16^^,^^54^^,^^194^ and clinical trials^5^, ^6^, ^7^, ^8^^,^^195^ following systemic VV injection. It should be noted that the lesions resolve without sequelae and do not require the interruption of the treatment. However, in some cases, mucocutaneous pustules contain infectious VV, indicating that the off-target skin infection may contribute to the environmental viral shedding.^7^^,^^195^ Similarly, a local injection of oncolytic HSV strains may lead to skin lesions and transient virus shedding, especially in seronegative patients.^196^^,^^197^ Dermal tropism is natural for both herpesviruses and poxviruses. Additionally to keratinocytes, these viruses may also target other cells in the skin. Thus, VV infects subdermal and dermal DCs, and the expression of early viral genes may influence subsequent antigen presentation to bystanders.^198^ Moreover, a recent study reports that after injection into cutaneous tumors, T-VEC enters and propagates not only in malignant cells but also in monocytes, macrophages, lymphocytes, and DCs.^199^ The authors conclude that the therapeutic efficacy of T-VEC is not solely based on selective direct oncolysis and that the infection of non-malignant cells is an important factor in reshaping the TME. Intriguingly, Minev et al. have shown that patients with VV-associated skin rashes demonstrate a trend toward longer survival.^200^ Collectively, these data suggest that off-target OV replication in the skin could play dual roles in cancer therapy.

There are many parallels in the microenvironment of solid tumors and healing wounds, including activation of neoangiogenesis, enhanced vessel permeability, and anti-inflammatory milieu.^138^^,^^201^ These similarities likely explain the ability of OVs to infect non-cancer cells in the sites of tissue repair. *In vitro* wound healing tests reveal that HUVECs at the leading edge of a scratch in the cell monolayer are preferentially infected by VV.^11^^,^^12^ *In vivo*, VV infection of ECs is found in wounded tissues, but not in uninjured skin.^11^ Consistently, the early stages of hepatic regeneration after resection provide an environment suitable for oncolytic HSV replication, leading to severe toxicity in mice.^202^ Presumably, induction of angiogenesis is also responsible for OV replication in the ovarian follicles, as shown for VV^18^, ^19^, ^20^, ^21^ and mumps virus.^203^ To the best of our knowledge, the consequences of OV infection in the spots of physiological neoangiogenesis have not been addressed yet.

## Concluding remarks

The overview of biodistribution studies demonstrates that multiple non-cancer cell types permit OV infection to some degree. Despite the high variability of infected non-cancer cells between different viruses and organs, some consistent patterns of this phenomenon can be concluded. First, there are virus-specific targets (e.g., liver for Ad, heart for reovirus) and cells/organs that are infected by OVs belonging to different families (e.g., tumor stromal cells, secondary lymphoid tissues). The former could be a serious concern for the therapies based on a certain viral vector, whereas the latter are even more important, reflecting some general principles of the virus-host interactions relevant to the whole field of OVT.

Second, from the mechanistic point of view, OVs target the organs that naturally accumulate a high amount of the virions (e.g., liver, spleen, lung, lymph node) or the spots of neoangiogenesis (tumor, wound, ovarian follicles). High virus to cell ratio may allow OVs to overcome intact antiviral response in normal cells, and *in vitro* studies support this possibility.^78^^,^^83^^,^^103^^,^^104^^,^^204^ Various concepts for OV detargeting from the cells of the reticuloendothelial system have been designed to prevent off-target infection and improve virus delivery to the tumor bed. In contrast, there is no strategy so far that aims to prevent OV replication in the spots of normal neoangiogenesis. To ensure the safety of OVT in the different clinical situations, the potential hazards of viral infection in healing wounds, ovarian follicles, placenta, etc. should be evaluated.

Third, the infected non-tumor targets can be classified into normal (e.g., macrophages, DCs, B cells, hepatocytes) and cancer-associated cells (e.g., tumor ECs, CAFs). The latter are reprogramed to promote a cancer-favoring microenvironment and therefore viral infection of these cells is likely beneficial for cancer therapy. Whether or not the infection of normal cells plays any role in OVT is not established yet.

From the prospect of virus-host interaction, OV infection of non-malignant cells may play dual roles. It can be regarded as the ability of the pathogen to break through host antiviral responses under certain conditions. It should be emphasized, though, that infection of normal cells is self-limiting and most likely does not contribute to the virus dissemination, whereas cancer cells are much more sensitive to OV replication and spread. Alternatively, anatomically restricted and time-controlled replication of the virus could be beneficial for a host, helping to develop a rapid proinflammatory response and activate the adaptive immune system. This mechanism is functional in the secondary lymphoid organs and could be extrapolated to other tissues. Thus, a wound is a potential site of entry for the microbes and the rapid activation of innate and adaptive immunity may play a critical role in antiviral defense. It is tempting to speculate that, by increasing the amount of the viral antigen in OV-permitting cells, more effective activation of IFN-producing and antigen-presenting cells can be achieved in the wound or tumor.

The ability of OVs to stimulate antitumor immunity is crucial for the success of the therapy and probably more impactful than direct lysis of the cancer cells. In this regard, the role of non-tumor cell infection in the complex interplay between antiviral and antitumor responses is of special interest. It cannot be excluded that, by activating rapid antiviral response, infection of normal cells limits OV replication in cancer cells and therefore interferes with the development of antitumor response. The other possibility would be that activation of the immune cells by transiently replicating viruses breaks the immune suppression established by the growing tumor. These hypotheses need to be tested to improve the efficiency of OVT by either enhancing or preventing normal cell infection.

While the intact immune system is able to control OV replication in normal cells, immunodeficiency may unleash the infection. It should be taken into consideration when OVs are co-administered with immunosuppressive drugs such as cyclophosphamide, paclitaxel, and cisplatin. Although these approaches demonstrate the improved antitumor responses in preclinical models,^39^^,^^77^^,^^87^^,^^205^, ^206^, ^207^ very few reports address the effects of chemotherapy on virus replication in normal tissues. Not surprisingly, cyclophosphamide increases the titers of MV in the spleen^39^ and reovirus titers in the heart.^77^ Even upon i.t. injection, reovirus titers are increased in the hearts of mice receiving cisplatin and paclitaxel.^87^ To ensure the safety of the combination therapy, the evaluation of OV replication in healthy tissues should become an integral part of the preclinical studies.

The infection of the non-tumor cells by OVs has another potential application. The ability of leukocytes to uptake the virus and migrate to tumors makes them an attractive vehicle for delivering OVs, especially in the case of pre-existing antiviral immunity.^208^ *In vitro*, by using high viral doses, a productive infection can be achieved in various normal cells, including monocytes,^78^^,^^107^ DCs,^83^^,^^103^^,^^104^ neutrophils.^204^ However, the role of virus replication in cellular vehicles is controversial. While productive infection multiplies the viral burden that can potentially be delivered,^209^ it may also lead to cell lysis and/or immune-mediated clearance of the vehicle before reaching the tumor.^84^ Understanding the mechanisms responsible for OV replication in normal cells and the fate of the infected cells *in vivo* will help improve the efficiency of cell-based virus delivery to tumors.

In summary, the current review demonstrates that OV infection of non-cancer cells is a widespread and yet underestimated phenomenon. A better understanding of the mechanisms responsible for normal cell infection is needed to ensure safety and potentiate the efficacy of OVT.
